# Oral health status, treatment needs and oral health-related quality of life in patients with epidermolysis bullosa in Austria: a mixed-methods pilot study

**DOI:** 10.3389/froh.2026.1826332

**Published:** 2026-05-04

**Authors:** Sebastián Véliz, Sophie Strobl, Hady Haririan, Wolfgang Manschiebel, Patricia Soare, Gudrun Salamon

**Affiliations:** 1 Department of Periodontology, Dental Clinic, Sigmund Freud University, Vienna, Austria; 2 Facultad de Medicina y Odontoloxia, Universidad de Santiago de Compostela, Santiago de Compostela, Spain; 3 Facultad de Odontología, Universidad de Chile, Santiago, Chile; 4 HEALTH Lab, Competence Centre for Medical and Health Psychology, Faculty of Psychology, Sigmund Freud University Vienna, Vienna, Austria; 5 Department of Dermatology, Medical Centre, Faculty of Medicine, University of Freiburg, Freiburg, Germany; 6 Dental Clinic, Sigmund Freud University, Vienna, Austria

**Keywords:** epidermolysis bullosa, oral health-related quality of life (OHRQoL), oral health assessment, dental treatment needs, mixed methods

## Abstract

**Introduction:**

Epidermolysis bullosa (EB) is a rare genetic disease with skin and mucosal fragility. Severe cases present major physical and psychological compromise, contributing to comorbidities and reducing oral health-related quality of life (OHRQoL). Despite a multidisciplinary oral healthcare approach is recommended for EB patients, there is not a specific pathway available in Austria. This study aims to explore the oral health status, oral health treatment needs and OHRQoL in Austrian patients with EB.

**Materials and methods:**

A cross-sectional, mixed-methods and exploratory study with purposive sampling was conducted, combining clinical assessment with quantitative and qualitative data by using standardized questionnaires, including OHIP-14, COHIP, iscorEB-p, QOLEB, and open-ended questions. Extraoral and intraoral assessments were conducted, including PhOX as well as oral treatment needs. Quantitative data were analysed descriptively, while qualitative data were subjected to reflexive thematic analysis to explore recurring patterns and themes in participants' experiences.

**Results:**

A total of 14 participants with genetic diagnosis of EB were included: EB simplex (EBS, *n* = 3) and dystrophic EB (dominant, DDEB, *n* = 1; and recessive, RDEB, *n* = 10). Participants living with RDEB exhibited poorer quality of life results (iscorEB-*p* = 39.4; QOLEB = 15.6), across all indexes compared to EBS (iscorEB-*p* = 17.3; QOLEB = 10.3), as well as worse oral health parameters and higher dental treatment needs. Most participants required preventive (84.6%), restorative treatment (76.9%), or speech therapy (69.2%). Compromised oral function was an important factor in QoL results and oral health assessment, but its improvement was not necessarily corresponding to oral treatment needs.

**Conclusion:**

EB, particularly RDEB, has a significant impact on oral health and OHRQoL in Austrian participants. Compromised oral function strongly influences outcomes, but improvements do not always match treatment needs. This underscore the need for multidisciplinary management, including speech therapy, to improve their OHRQoL.

## Introduction

Epidermolysis bullosa (EB) is a group of genetic disorders characterised by skin and mucosal fragility, caused by variants in the genes encoding proteins of the dermal-epidermal adhesion zone (1, 2). The current classification considers four major types: EB simplex (EBS), Junctional EB (JEB), Dystrophic EB (DEB) and Kindler EB (KEB). These types are further subdivided into subtypes based on the molecular findings, since distinct genetic variants can cause similar phenotypes; and the same gene, due to the inheritance trait, can result in different phenotypic expression, such as dominant (DDEB) or recessive (RDEB) dystrophic EB (1). Different reports have shown a wide range of prevalence per 1 million inhabitants, ranging from 4.4 in Romania (3) to 54 in Germany (4). In Austria, it has been estimated that around 500 people live with EB (5).

The main clinical feature of EB is the presence of skin and mucosal lesions, such as erosions, vesicles and blisters, caused by low-intensity mechanical forces. Other manifestations will vary according to the type and subtype and can include milia, skin and nail atrophy, granulation tissue, palmoplantar keratoderma, mottled pigmentation and pigmented naevi, scarring and contractures. Secondary manifestations can compromise other organs or functions, including but not limited to the eyes, ears, oesophagus and gastrointestinal tract (2).

Due to the fragility of the mucosa, various orofacial manifestations may occur according to the type and subtype of EB. Most patients, including those with low and moderate risk of oral disease, such as EBS and DDEB, can present mucosal lesions, such as bullae, blisters, ulcers or erosions (6, 7). Additionally, patients with a high risk of oral disease can present specific features (6). Patients with JEB often present perioral granulation tissue, gingival hyperplasia and syndromic amelogenesis imperfecta with different phenotypes according to the affected genetic variants, with associated crown resorption, delayed eruption, agenesis, and attrition (6–9). People with severe subtypes of RDEB, such as RDEB severe, intermediate or inversa, can present extreme mucosal fragility with recurrent lesions, oral strictures such as microstomia, ankyloglossia and vestibule obliteration; mucosal atrophy with absence of tongue papillae and palatal rugae; poor oral hygiene due to oral and hand strictures leading to higher gingival and caries scores; higher prevalence of malocclusions and oral cancer (6, 7, 10–14). KEB, the rarest form of EB, presents a high prevalence, early onset and fast progression of periodontal disease, syndromic amelogenesis imperfecta, and oral strictures including microsomia and vestibule obliteration as well as in some cases oral squamous cell carcinoma (6, 7, 15–17).

The broad phenotypic expression of oral features EB makes that the patients require different specialist services according to their needs (7). Comprehensive dental care has been suggested to start from an early age, tailored to their individual risk of oral disease, since early intervention and prevention can alleviate the oral health burden (6). Although dental treatment needs have been studied in children, with most patients requiring preventive and restorative treatment, evidence in adults remains scarce (18). Therefore, integrating oral health care in the multidisciplinary management of patients with EB is crucial, as it impacts nutrition, speech and QoL (6). Regardless of the considerable efforts made be DEBRA Austria to improve the life of those living with EB in the country, there is no implemented EB oral health care pathway in Austria. Therefore, people living with EB in Austria must organise their own oral healthcare within the existing healthcare system. Although Austria hosts one of the largest EB centres in the world, a systematic assessment of the oral health characteristics and needs of this population is considered a relevant research gap (19).

Quality of Life (QoL) is significantly compromised in people with EB affecting their personal, physical, emotional and professional life (20). This also includes their Oral Health-Related Quality of Life (OHRQoL) (21–23). Fortuna (2016) found that DEB patients showed impairments across all OHIP-49 dimensions, with the greatest limitation in “functional limitation” and “physical disability”. However, these impairments of OHRQoL did not correlate with the number of affected oral-pharyngeal sites involved and oropharyngeal disease severity as measured by the Epidermolysis Bullosa Oropharyngeal Severity (EBOS) score (23, 24). This discrepancy underscores the need for further research, that integrates clinical findings and patients-reported experiences.

In children, it has been documented that patients living with DEB have a significant compromise in OHRQoL, using the COHIP and a qualitative accounts, showing how patients cope with the disease (21). Despite this, further research is needed to better inform dental care strategies at improving OHRQoL in people living with EB. This research aims to explore the relation between oral findings and dental treatment needs with their QoL/OHRQoL in people living with EB in Austria.

## Materials and methods

A cross-sectional, mixed-methods and exploratory study was conducted in January 2025 at the Department of Periodontology of the Dental Clinic of Sigmund Freud University (SFU), in association with DEBRA Austria. The study comprised a two-stage assessment: QoL assessment, including qualitative data analysis and validated questionnaires monitored by the SFU's HEALTH Lab, and an orofacial examination. A multidisciplinary team of dental professionals, an EB oral health specialist and psychologists specialising in EB were involved in each step of the study.

A purposive sampling strategy was employed to achieve a high range of oral manifestation and its degree of severity, age and gender. Participants were recruited in cooperation with DEBRA Austria based on the following inclusion criteria: a diagnosis of any EB type, resided in Austria, consented to participate, and were able to attend the Dental Clinic of SFU for oral assessment. Exclusion criteria were failure to meet the inclusion criteria and not following instructions during the examination. Written informed consent or assent was obtained from all participating patients, or from legal guardians of underage patients. For underage patients, quantitative data was obtained via a parent or guardian acting as a proxy. During the examination, self-report was supplemented with accounts from parents or guardians if needed. This research was approved by the Ethics Committee of the Sigmund Freud University, Vienna, Austria (EK: 801-2023) and funded by the Austrian Dental Society (Österreichische Gesellschaft für Zahn-, Mund- und Kieferheilkunde, ÖGZMK). This report was written in accordance with the Strengthening the Reporting of Observational Studies in Epidemiology (STROBE) guideline for cross-sectional studies (25).

### Quality of life assessment

A mixed-methods approach was implemented to assess OHRQoL and EB-QoL by means of an online survey completed prior to the examination. In addition to sociodemographic data, open-ended questions concerning EB-OHRQoL standardized questionnaires on the patients' QoL, dental and general health status were implemented. The included questionnaires were the Oral Health Impact Profile (OHIP-14, German version) (26), Child Oral Health Impact Profile (COHIP-19, German version) (27), Instrument for Scoring Clinical Outcomes of Research for EB (iscorEB-p) (28) and the Quality of Life in Epidermolysis Bullosa (QOLEB, German version) (29). The qualitative data were supplemented with open questions on their oral health and QoL during the online questionnaires, and with field notes collected during the orofacial assessment. Additionally, for the EB diagnosis and the degree of severity, information was obtained from genetic testing and external assessments by attending physicians at EB House Austria, respectively.

### Orofacial assessment and treatment need

The dental assessment was performed by a team of trained dentists accompanied by an EB oral health trained specialist at the Department of Periodontology of the Dental Clinic of SFU, using as reference the Epidermolysis Bullosa—Oral Health Assessment Form (EB-OHAF) from the Faculty of Dentistry of the University of Chile and, in those with complete permanent teeth, the Physical Oral Health Index (PhOX) (30). A panoramic radiograph was performed on the day of examination for all patients.

The description of each assessed variable is included in the Supplementary Appendix 1 and covers the presence/absence of extraoral and intraoral lesions, mouth opening, vestibule depth, tongue protrusion, tongue texture, palatal texture, community periodontal index, the Decay-Missing-Filled Teeth Index (DMFT), the Oral Hygiene Index (OHI), malocclusion and oral function impairment. Community Periodontal Index was assessed in each patient using a periodontal probe (PCP12, Hu-Friedy®). The treatment needs were established according to the results of the orofacial examination in line with the criteria outlined in Supplementary Appendix 2.

### Risk of bias

To decrease the risk of bias, the dental team was provided with special training by the EB oral-health specialist, who detailed each potential diagnosis to mitigate any potential bias during the examination. Furthermore, all examinations were conducted by a single dentist (HH) and the EB oral-health specialist (SV). In the event of uncertainty, a consensus meeting was held, including the whole team. The information was anonymised and then analysed jointly by dentists and psychologists.

### Qualitative and statistical analysis

A descriptive statistical analysis was performed to present the characteristics of this cohort, including percentages, means, medians, and standard deviations. All statistical calculations were performed using IBM SPSS Statistics software, version 29. Missing data were directly addressed in the Tables 1, 2. Qualitative data were analysed by means of thematic analysis (31).

**Table 1:** Sociodemographic characteristics of the sample.

Sample Size		14 participants	
Average age		23.5 (SD = 19.8, MD = 16.5)	
		Range from 2 to 70 years	
		*participants (n)*	*percent*
Gender	Female	7	50.0%
	Male	7	50.0%
EB type (genetic testing)	EBS	3	21.4%
	DDEB	1	7.1%
	RDEB	10	71.4%
EB subtype (genetic testing)	Generalized	1	7.1%
	Inversa	1	7.1%
	Intermediate	4	28.6%
	Severe	7	50.0%
	Missing	1	7.1%
EB severity (physician's assessment)	Mild	5	35.7%
	Moderate	2	14.3%
	Severe	7	50.0%
EB visibility	Very	5	35.7%
	Somewhat	5	35.7%
	Rather not	4	28.6%
EB affected areas	Hair	4	28.6%
	Face	5	35.7%
	Eyes	6	42.9%
	Mouth	10	71.4%
	Oesophagus	8	57.1%
	Neck and shoulders	4	28.6%
	Hull	4	28.6%
	Genital	4	28.6%
	Arms & hands	8	57.1%
	Legs & Feet	11	78.6%
Toothache	Never	7	50.0%
	Rarely	1	7.1%
	Sometimes	4	28.6%
	Often	2	14.3%
Restrictions on eating	Never	4	28.6%
	Rarely	4	28.6%
	Sometimes	2	14.3%
	Often	4	28.6%
Halitosis	Never	7	50.0%
	Rarely	5	35.7%
	Sometimes	0	0%
	Often	2	14.3%

EB, epidermolysis bullosa; EBS, EB, simplex; DDEB, dominant dystrophic EB; RDEB, recessive dystrophic EB; SD, standard deviation; MD, median.

**Table 2:** Clinical characteristics of the sample.

Clinical characteristics		Participants (*n*)	Percent
Importance of oral health	Very important	11	78.6%
	Important	2	14.3%
	Rather not important	1	7.1%
	Not important	0	0%
Frequency dental visits	<once a year	4	28.6%
	Once a year	6	42.9%
	>once a year	4	28.6%
Causes for dental visits	Routine check-ups	8	57.1%
	Acute issues	6	42.9%
Satisfaction with availability of dental care	Very satisfied	1	7.1%
	Satisfied	11	78.6%
	Dissatisfied	1	7.1%
	Very dissatisfied	1	7.1%
Contentment with dental care	Very satisfied	3	21.4%
	Satisfied	9	64.3%
	Dissatisfied	1	7.1%
	Very dissatisfied	1	7.1%
Traumatic or negative Experiences due to complications during or after dental treatment		4	28.6%

## Results

### Study participants

A total of 24 participants were initially contacted and asked to take part in the study. Of these, 15 agreed, and 14 were successfully recruited. First, QoL assessment was obtained (*n* = 14). Regarding the orofacial assessment, one participant was assessed externally only due to their young age (*n* = 13) (Supplementary Figure S1).

Data on a total of *n* = 14 patients living with EB in Austria was collected, whereof half of the participants' survey data was collected via report by proxy (i.e., their parents) (*n* = 7; 50%). Participation was equal for males (*n* = 7, 50%) and females (*n* = 7, 50%), with an average age of 23.5 years (SD: 19.85; MD = 26.50; range: 2-70). Regarding the distribution of the EB types within the sample, 3 (21.4%) were diagnosed with EBS, 1 (7.1%) with DDEB and 10 (71.4%) with RDEB. The visibility of EB was evenly distributed, whereas half of the participants (50%) were estimated to have a severe form of the disease. More than a third of the sample (71.4%) stated that EB affects their mouth. Symptoms reported included toothaches, halitosis and eating restrictions. The distribution of EB-affected areas is illustrated in Table 1.

### Experiences with dental care in Austria

Regardless of EB type, most participants considered oral health “very important” (*n* = 11, 78.6%) or “important” (*n* = 2, 14.3%), with only one participant considering it “not so important” (*n* = 1, 7.1%). The majority attend the dentist at least once a year (*n* = 10, 71.4%), with routine check-ups being the most common reason (57.1%) (see Table 2), resulting in 62.3% visiting the dentist often and being content (See Table 3).

**Table 3:** Frequency of overall support use and contentment (in percentages).

Contentment	High Frequency	Low Frequency
**Contentment**	**62**.**3**	**21**.**5**
**Discontentment**	**7**.**1**	**7**.**1**

A total of 12 patients (85.7%) reported being satisfied or very satisfied with their dental treatment in the quantitative survey, citing the dentist's patience and care being the most common feedback. (See Table 3).

“My current dentist and assistant are aware of my EB problem. They take their time and leave enough time between treatments for healing.” (N°13, RDEB)

On the contrary, the main problem reported during the examination was the lack of an EB-trained specialist in their area. Being treated by dentists who are unaware of the condition and its effect on dental and oral care often results in wounds and blisters emerging after treatment.

“Most dentists are unfamiliar with EB and often do not listen when something is explained to them, resulting in them acting inappropriately and causing significant injury.” (N°11, RDEB)

“'Whenever I go to the dentist, this part [the inside of his cheek] is ripped open.” (N°12, RDEB)

One patient even reports that, despite mentioning acute pain, the dentist repeatedly delays treatment to avoid causing further damage because of EB.

“Because [name of dentist] always tends to wait longer and not treat immediately, which once led to me having to go to the emergency clinic and receive emergency treatment after weeks of pain. And I think it’s starting again up there—I've said several times that I'm in pain, and she just said, ‘That’s fine’, and it’s not.” (N°11, RDEB)

Almost a third of patients (28.6%) had experienced negative or traumatic situations due to complications during or after dental treatment (see Table 3). Negative experiences are reactivated during subsequent visits, causing anxiety before treatment begins and negatively affecting mental health. Patients delay examinations for as long as possible. Two patients stated that they are now only willing to undergo treatment with nitrous oxide support or under general anaesthesia. This is even more difficult with children. One mother reports that her daughter simply refuses to open her mouth during dental appointments out of fear.

“Once something went really wrong, and now every time I go to the dentist, I can smell it and it brings back memories. Another dentist then did something wrong, and that was it. Now I only go when it’s absolutely necessary.” (N°3, EBS)

“They said a lot of teeth need to be removed, they said there’s tooth decay. [Name of child with EB] is very scared and doesn't want to do it, she won't open her mouth.” (N°8—Mother, RDEB)

Additionally, two of the patients (14,2%) were dissatisfied with the availability of dental care.

“Geographical conditions: the special clinic is more than two hours away by train, so a visit to the dentist requires at least one day away from school and an overnight stay away from home.” (N°7, RDEB)

### OHRQoL assessment

All RDEB participants (*n* = 10, 100%) report that most oral health problems are related to functional limitations, including chewing, swallowing and speaking. While some participants report no restrictions in the mouth area, others report painful oral lesions and/or strictures, recurring blisters, sores, and gum inflammation.

“My gums are always a bit inflamed—I also have bleeding gums from time to time.” (N°12, RDEB)

“This causes me regular problems because the pulling affects my lip and I regularly get a blister because of the pulling—even when I yawn, it hurts.” (N°11, RDEB)

For some participants, the relation between their nutrition and pain seems obvious, i.e., pain when consuming cold liquids or being exposed to heat, other patients feel confronted with the symptoms without understanding their origin.

“On my tongue I have blisters regularly I don’t know if it comes from specific food or if it is just there. When I eat an apple or chips I can feel the wounds developing.” (N°11, RDEB)

“For months, I've been experiencing a bit more pain when drinking cold liquids, and I'm very sensitive in that area. I think it’s the front tooth because that’s exactly where I feel it when I brush my teeth and when eating certain foods.” (N°11, RDEB)

“But she is sensitive to the heat.” (N°7, RDEB)

“Sometimes it bleeds a little. She sometimes gets blisters from eating, but that doesn't usually bother her. Only when they get bigger do they hurt, and then we pierce them.” (N°5, RDEB)

Meals are adapted as necessary, with harder foods often being avoided or at least chewed carefully. Some participants reported that several oesophageal dilations were required to facilitate swallowing, a process described as a psychological burden.

“A little—but not that it affects my diet—regular food, but I must chew very carefully that I don’t swallow big pieces.” (N°11, RDEB)

“She has a little difficulty eating—she currently only eats purée and milk. She also eats bananas.” (N°14—Mother, RDEB)

“I always struggle to swallow, but I can take very small tablets. The same problem occurs after eating. When I'm not at home, I always have water to rinse with.” (N°13, RDEB)

“We used to have, we had like 3 bundled procedures in Salzburg [oesophageal dilations between the ages of 4 and 6].” (N°7—Mother, RDEB)

“Last time [name of patient] had problems swallowing. Once a year, the oesophagus is stretched. It is a psychological burden to travel there [Salzburg].” (N°9, RDEB)

Limited dietary options, as well as visible restrictions, are perceived as impairments and also affect social behaviour, leading to feelings of shame and social withdrawal.

“My oral health has a strong impact on my behaviour. I try to keep my mouth closed when I speak or smile. Eating in public is difficult because I often choke due to the narrowing of my oesophagus.”(N°13, RDEB)

In regard to daily dental care, several participants described brushing their teeth as a challenge. Brushing habits varied, with some preferring electric ultrasonic toothbrushes and others relying on manual toothbrushes. These differences often reflected the difficulty of finding ways to minimise pain, irritation, or mucosal injury during daily oral hygiene.

“She is doing it on her own and sometimes I am doing it after because she says she cannot reach a location but basically she is doing it on her own” (N°7—Mother, RDEB)

“Since the electric toothbrush—The dentist says it’s much better than before” (N°11, RDEB)

“We tried electric toothbrushes, realized that they didn't work so well, and went back to manual toothbrushes because they work better and leave less plaque behind.” (N°1—Father, EBS)

Adults and teenagers over 12 years old (*n* = 10) were asked to complete the OHIP-14. The mean value for the sample was 13.3 (SD: 9.02, MD: 13), with the lowest value of 5 observed in the patient with DDEB, followed by EBS (Av: 9; SD 11.3; MD: 9), and the highest mean in the RDEB participants (X = 15.7; SD: 8.7; MD:14), suggesting that participants with EBS have a higher OHRQoL.

“Not such a problem in the mouth. Mhm, definitely open sores, but no blisters.” (N°4, DDEB)

The same interpretation was obtained for the COHIP, used with the 7 participants of 18 years or younger (EBS = 2, RDEB = 5). The mean value for the group (*n* = 7) was 58.1 (SD: 24.9; MD57), with a higher mean for the EBS group of 69.5 (SD: 6.3, MD: 70) when compared to the mean of 53.6 (SD: 15.8; MD:55) of the RDEB group. For specific EB instruments, mean values were also indicative of lower health status (iscorEB-p) and QoL (QOLEB) in participants with RDEB (iscorEB-p = 39.4—SD:20.1—MD:34; QOLEB = 15.6—SD:7.7—MD:14.5), when compared to DDEB (iscorEB-p = 10; QOLEB = 5) and EBS (iscorEB-p = 17.3—SD:7.5—MD:14; QOLEB = 10.3—SD:5.1—MD:9). Values for QoL indexes are shown in Table 4.

**Table 4:** OHRQoL and EB-QoL results.

General data		QoL																						
ID N°	EB type	OHIP-14								COHIP-19				iscorEB-p								QOLEB		
		Functional limitation	Physical pain	Psychological discomfort	Physical disability	Psychological disability	Social Disability	Handicap	TOTAL OHIP-14G	Oral health/well-being	Functional well-being	Social/emotional, school and self-image	Total COHIP-19	Pain	Itching	Essential functions	Sleeping	Daily activities	Mood	Impact	Total iscorEB-p	Functioning	Emotions	Total QOLEB
1	EBS	/	/	/	/	/	/	/	/	14	15	36	65	6	2	0	0	2	2	0	12	4	5	9
2	EBS	0	0	0	0	0	0	1	1	19	16	39	74	6	2	0	0	2	2	2	14	10	6	16
3	EBS	3	3	2	1	0	4	4	17	/	/	/	/	14	0	0	4	0	0	8	26	5	1	6
4	DDEB	0	1	0	1	0	1	2	5	/	/	/	/	8	2	0	0	0	0	0	10	4	1	5
5	RDEB	/	/	/	/	/	/	/	/	20	16	40	76	6	4	4	2	0	2	0	18	4	3	7
6	RDEB	/	/	/	/	/	/	/	/	11	9	24	44	24	4	4	0	12	4	8	56	16	6	22
7	RDEB	0	2	3	4	1	1	1	12	14	10	35	59	4	2	2	2	4	2	12	28	11	6	17
8	RDEB	0	4	4	4	2	4	3	21	7	8	19	34	24	4	4	0	12	4	8	56	16	6	22
9	RDEB	0	0	0	0	0	0	2	2	/	/	/	/	16	6	2	0	6	0	2	32	8	4	12
10	RDEB	4	4	4	4	4	4	2	26	/	/	/	/	30	6	16	6	10	4	10	82	18	13	31
11	RDEB	0	2	4	2	0	0	2	10	/	/	/	/	10	6	4	0	2	4	0	26	10	5	15
12	RDEB	3	3	3	1	1	2	1	14	/	/	/	/	6	4	4	2	0	0	2	18	4	2	6
13	RDEB	4	4	4	3	1	5	4	25	/	/	/	/	18	4	6	2	2	2	2	36	6	3	9
14	RDEB	/	/	/	/	/	/	/	/	18	9	28	55	18	6	4	6	2	2	4	42	11	3	14

EB, epidermolysis bullosa; EBS, EB simplex; DDEB, dominant dystrophic EB; RDEB, recessive dystrophic EB, M, male; F, female.

### Orofacial assessment and treatment needs

**Figure 1:**
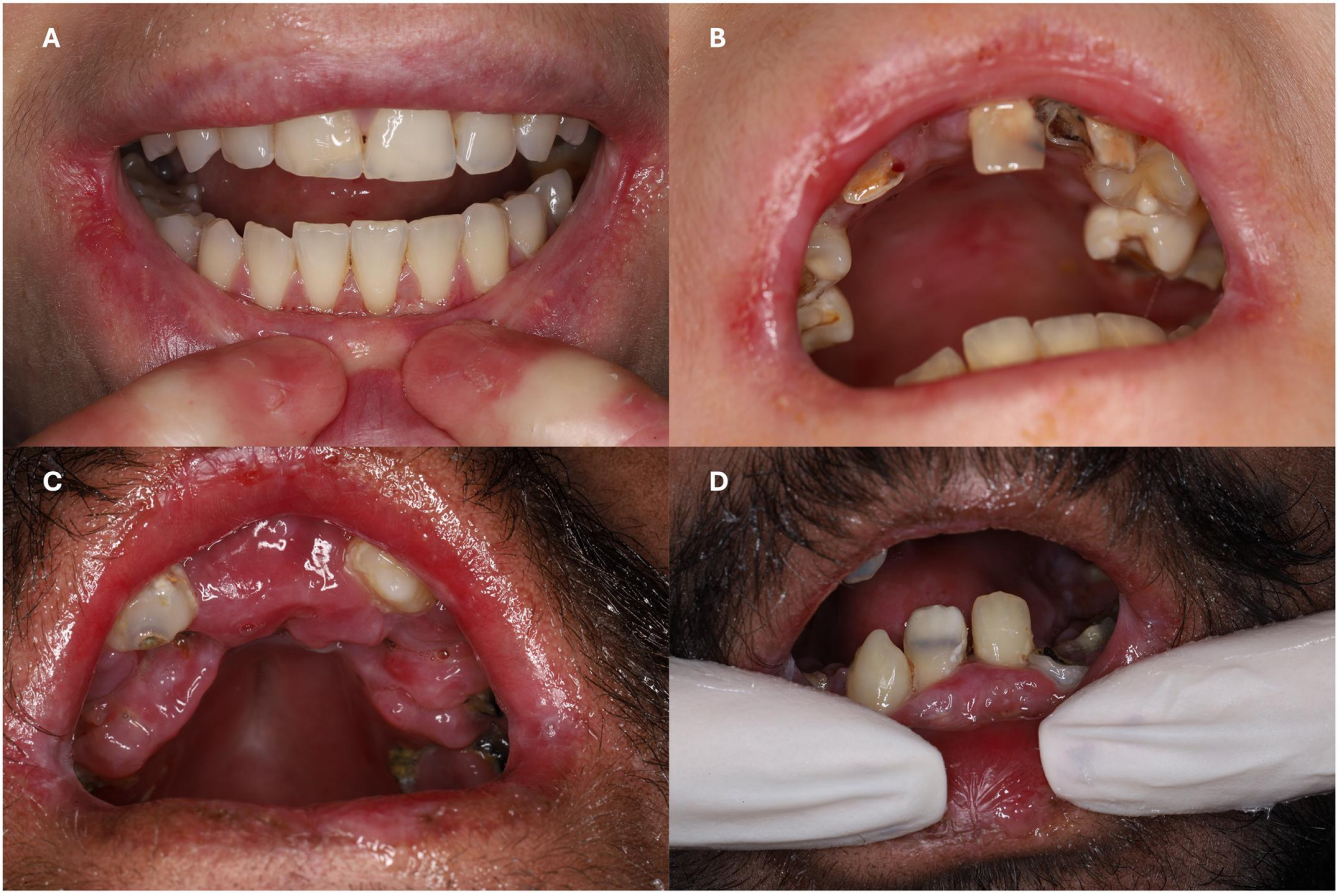
Clinical photographs of participants with recessive dystrophic epidermolysis bullosa (RDEB). **(A)** Participant with Intermediate RDEB presenting relatively good oral health and predominantly healthy condition. **(B–D)** Participants with severe RDEB presenting severe microstomia, gingival inflammation, extensive dental caries and multiple residual roots. Photos C and D are from the same participant.

All participants with EBS (*n* = 3/3) and DDEB (*n* = 1/1) showed no intraoral lesions, strictures (microstomia, ankyloglossia or vestibule obliteration), malocclusion or compromised oral function. On the contrary, all participants with RDEB (*n* = 9/9, 100%) presented any type of intraoral lesion, ankyloglossia, compromised tongue texture (either partially or totally depapillated), and oral function impairment (Figure 1). These functional limitations often were reported to hurt.

“When there’s a sore spot and I swallow, it hurts.” (N°8, RDEB)

“Sometimes I have pain in my mouth” (N°10, RDEB)

“So, when I didn't have the smooth crowns yet, everything was open.” (N°13, RDEB)

In adolescents and adults with complete permanent teeth, PhOX values show an average of 59.6 ± 17.2 for the whole group (*n* = 10), ranging from 94 (Participant N°2, EBS) to 24 (Participant N°10, RDEB). EBS participants presented a higher optimal oral health, with a higher average score (80.5 ± 19.9, *n* = 2) than DDEB (61, *n* = 1) and RDEB (53.2÷13.8, *n* = 7), with worst physical oral health. The oral characteristics observed in this cohort are shown in detail in Table 5.

**Table 5:** Oral health status and dental treatment needs results.

General data		Dental treatment																						
ID N°	EB type	Orofacial assessment																Treatment need summary						
		Extraoral lesions	Intraoral lesions	Mouth opening (mm)	Vestibule depth (mm)	Tongue protrusion (mm)	Tongue texture	Palatal texture	BPE (Highest)	DMFT/deft	Decay	Missing	Filled	OHI	Malocclusion	Orofacial function impairment	PhOX	Preventive	Periodontic	Restorative	Orthodontic	Prosthodontics/ Implantology	Surgery	Speech therapist
1	EBS	No	No	42	9.1	25	Normal	Normal	2	0/1	0/1	0	0	2	No	No	/	Yes	No	Yes	No	No	No	No
2	EBS	No	No	40	9.8	21	Normal	Normal	2	0	0	0	0	1.3	No	No	94	Yes	No	No	No	No	No	No
3	EBS	No	No	47	11.4	28	Normal	Normal	4	16	5	4	7	1.5	No	No	67	No	Yes	Yes	No	Yes	No	No
4	DDEB	Yes	No	62	13.3	38	Normal	Unusual	2	22	2	10	10	2	No	No	61	Yes	No	Yes	No	Yes	No	No
5	RDEB	No	Yes	20	4.2	3	Depapillated	Normal	/	-/0	0	0	0	/	No	Yes	/	Yes	No	No	No	No	No	Yes
6	RDEB	No	Yes	18	0	2	Depapillated	Absent	/	3/5	3/5	0	0	3	Yes	Yes	/	Yes	No	Yes	Yes	No	No	Yes
7	RDEB	Yes	Yes	15	0.2	0	Depapillated	Unusual	1	4	0	4	0	2.3	Yes	Yes	65	Yes	No	No	Yes	No	No	Yes
8	RDEB	Yes	Yes	14	0.1	0	Depapillated	Normal	2	16	14	2	0	3	No	Yes	54	Yes	No	Yes	No	Yes	Yes	Yes
9	RDEB	Yes	Yes	17	0.9	1	Depapillated	Absent	2	10	1	9	0	2.5	Yes	Yes	50	Yes	No	Yes	No	No	No	Yes
10	RDEB	Yes	Yes	18	4.8	1	Depapillated	Absent	3	25	4	21	0	3	No	Yes	24	No	Yes	Yes	No	Yes	Yes	Yes
11	RDEB	No	Yes	35	6.7	16	Partially depapillated	Unusual	2	19	3	1	15	0.2	No	Yes	60	Yes	Yes	Yes	No	No	No	Yes
12	RDEB	No	Yes	31	8.3	2	Depapillated	Unusual	2	15	1	0	14	1.3	Yes	Yes	61	Yes	No	Yes	Yes	No	No	Yes
13	RDEB	No	Yes	46	9.8	7	Partially depapillated	Unusual	3	23	3	3	17	0	No	Yes	60	Yes	No	Yes	No	No	No	Yes
14	RDEB	Orofacial assessment was not possible															/	Orofacial assessment was not possible						

EB, epidermolysis bullosa; EBS, EB simplex; DDEB, dominant dystrophic EB; RDEB, recessive dystrophic EB; M, male; F, female. BPE, basic periodontal examination; DMFT, decay-missing-filled teeth index; OHI, oral hygiene index.

During the assessments several participants mentioned limitations in opening their mouth, resulting in difficulties during dental treatments.

“The dentist was never happy with my range [opens mouth]” (N°12, RDEB)

“My small mouth opening was always a problem during [dental] procedures” (N°13, RDEB)

“No, she can't (open her mouth) only halfway.” (N°14—Mother, RDEB)

This was also mentioned to be an important factor for dental assessment and operations, as one participant noted.

“I can even open it wider, when I exercise before and before an operation I usually exercise.” (N°11, RDEB)

Speech therapy was reported as a supportive measure to maintain oral function, particularly to improve oral function and the mouth opening.

“We usually did speech therapy before surgery to keep the mouth more open” (N°7—Mother, RDEB)

Regarding treatment needs, most patients require preventive (*n* = 11/13, 84.6%) and restorative treatment (*n* = 10/13, 76.9%), followed by speech therapy (*n* = 9/13, 69.2%), prosthodontics/implants (*n* = 4/13, 30.7%), orthodontics and periodontics (*n* = 3/13, 23.0%) and oral surgery (*n* = 2/13, 15.3%).

“I had a full set of braces when I was eight. I had surgery on my whole mouth, as they said it was too risky to do it every week. That was [more than 20 years] years ago and it was the biggest surgery. It was every few weeks at the dentist’s, and she said that I suffered every time, so let’s do it all at once, because I was only [age under 10].” (N°12, RDEB)

“We extracted the sixes at the back. They were perfectly healthy, but it’s standard practice in EB therapy to remove teeth as soon as possible. But then it’s kind of a preventive measure to take them out.” (N°7—Mother, RDEB)

All participants with RDEB require speech-language therapy (*n* = 9/9), with the impact of the wounds and blisters on the speech of those affected being mentioned.

“My husband has been saying for the last few days that I'm mumbling again—but it’s already starting to heel.” (N°13, RDEB)

“And then we had logopaedic training, and since then we didn’t have to” (N°7—Mother, RDEB)

In practice, prosthodontic treatment was found to be difficult to implement, as evidenced by the following statement from a patient:

“I had the prosthesis made because I thought it would be a relief, but there is just constant pressure on the tooth and the braces. It’s like pinching your finger, and I had it adjusted constantly, but then it got looser and looser and blisters started to form. It helps me eat, but it hurts so much” (N°3, EBS)

The number of specialities required ranged from 1 (Participant N°5, EBS, Preventive treatment) to 5 (Participants N°8 and 10, Preventive/Periodontics, respectively, Restorative, Prosthodontics/Implants, Surgery and Speech therapy). Additionally, only two patients presented chewing problems due to missing teeth (more than 4 missing teeth), with most RDEB participants having a history of oesophageal dilation and complaining about difficulties in eating due to functional limitations in swallowing. Other results are shown in detail in Table 5.

## Discussion

This mixed-methods pilot study showed the relation between oral health, oral treatment needs and oral health-related quality of life, in a cohort of participants living in Austria with different types of EB. Our results suggest that oral manifestations, specifically in RDEB, can affect oral health, oral function and OHRQoL, requiring more difficult dental treatments, with a higher number of specialists involved. In most cases, this must include a speech therapist, as orofacial function can significantly affect the OHRQoL, but will not necessarily be restored after dental treatment.

Over the past decades, intensive efforts by the patient organisation DEBRA Austria have created a unique support structure for affected individuals and their families, providing a wide range of services, including international reference for EB networks. Despite this, no EB-specific dental care pathway has yet been established in Austria. Previous studies have evaluated the relationship between oral health and QoL in adult population in Austria, but no EB studies related to oral health could been found by the authors (32). Therefore, the present article is the first attempt to address the impact of oral health in the OHRQoL for Austrian EB population.

The oral characteristics of this cohort are similar to previous evidence, as the participants with RDEB described here showed a higher prevalence of oral lesions, caries and malocclusions, with signs of oral atrophy (depapillated tongue, absence of palatal rugae) and severe strictures causing microstomia, vestibule obliteration and ankyloglossia (7, 11, 33, 34). PhOX values also correlate with previous evidence, as Joanning et al. reported a PhOX value for DEB of 54.6 ± 15.7, similar values to the ones observed in this study (22). Additionally, their oral function is affected, including deglutition, chewing, and swallowing limitations, as it has been previously stated that strictures limit oral functioning in patients with RDEB (33). Participants with EBS and DDEB presented fewer oral lesions and manifestations, consistent with the existing evidence (7). Although this research did not include participants with JEB or KEB; it is necessary to note that oral manifestations vary across EB types and subtypes.

According to the dental findings, treatment requirements included mostly preventive and restorative treatments, similar to the results reported by Smith et al. in children with EB (18). In this Austrian cohort, dental specialists needed included prosthodontics/implants (*n* = 4), periodontics and orthodontics (*n* = 3), and oral surgery (*n* = 2), which has been stated previously in patients with a high risk of oral disease (6). However, despite all patients with RDEB examined orally (*n* = 9) reporting functional limitations, only 2 would potentially benefit if receiving the proper dental treatment, as those with severe tooth loss would improve their chewing capacity after oral rehabilitation. This has been previously reported, with cases of patients living with EB who improved their ability to chew, swallow and OHRQoL after oral rehabilitation (7, 35–38). Still, in many regions, specialised treatment for EB patients is limited, particularly in remote areas. Therefore, increasing awareness among general dentist about EB, the main oral manifestations, and basic management principles is essential (39).

Oral health can significantly affect the QoL of patients with rare diseases such as EB. The effects on nutrition, chewing, speech and the accompanying social implications, as well as anxiety surrounding dental treatment and the consequent delay in treatment, impact QoL. Previous studies have shown that pain and the inability to chew can compromise the QoL in patients with rare diseases, with a higher impact in the domains of psychological discomfort, followed by functional limitation, social disability and physical pain (40). Similar results have been found in patients with EB. In Children, Marte et al., using a mixed-method methodology, reported that patients wish more consideration concerning oral health care, specifically oral function and aesthetics, emphasising the importance of early preventive measures (21). The need for clear communication was also reflected in our study as one participant asked:

“Please explain in simple terms, because I go to the dentist so often that I would like to understand many things better.” (N°11, RDEB)

For this study, QoL instruments were used including two for OHRQoL [OHIP-14, German version (26), COHIP-19, German version (27)] and two for EB (iscorEB-p (28), QOLEB, German version (29). All instruments show that RDEB patients experience the most severe impairments, compared to other EB groups. In contrast, the mean values of EBS participants indicate the least restrictions or negative effects of EB on their quality of life, which is also consistent with the information provided in the literature. Fortuna et al. focused on the limitations imposed on OHRQoL in DEB participants, comparing them with a control group, using clinical al QoL instruments such as the Oral Health Impact Profile (OHIP-49) (41), the RAND Short Form-36 (SF-36) (42), Hamilton Rating Scale for Anxiety (HAM-A) (43), Hamilton Rating Scale for Depression (HAM-D) (44) and Epidermolysis bullosa oropharyngeal Severity Score (EBOS) (24).The results showed that patients suffering from DEB have to live with significant limitations in the functional dimensions of the body compared to the control group and suffer from pain significantly more often. Although a positive correlation was found between poor oral health-related quality of life and psychological distress such as anxiety and depression, no significant association between general and oral health-related quality of life was found (23). The study by Joanning et al. correlated OHIP-14 and PhOX in adults living with EB in Germany. Both, OHRQoL and clinical oral health were most severely impaired in DEB patients (22). In Children, Marty et al. used the full version of the COHIP, analysing it by each domain, while in the present study, the short version (COHIP-19) was used. Despite this, their results show that people living with EB, especially RDEB, present a compromised QoL, with domains “Oral health” and “functional well-being” showing poor results, which is consistent with our results (21). This indicates that function is one of the main affected components in OHRQoL for people living with EB in Austria. Therefore, whenever possible, dental treatment should prioritise enhancing and/or rehabilitating oral function, as this is a primary concern for patients and has a significant impact on their OHRQoL. This should be done by a multidisciplinary dentomedical team that includes a psychologist to manage anxiety or fear of dental treatment, and a speech therapist because oral function is not always restored solely by dental treatment. Future interventions should aim to implement a dental care pathway for the EB population in Austria, that includes special care dentistry provided by a multidisciplinary team, as suggested by the Oral health care pathways for patients with EB from the European Reference Network for rare skin diseases (6).

Although a detailed analysis of the responses reveals statements and assessments of EB-associated impairments suggesting a high to very high level of suffering among patients, the overall mean values of the individual questionnaires consistently indicate moderately reduced quality of life related to oral health in Austria. This also was noted by Marty et al. in children, who despite presenting poor results in oral health and functional well-being, the qualitative analysis showed that the participants seemed to deal relatively well with their condition (21). In this regard, it must be noted that in the present sample, when asked how severe their EB was, only three of the patients reported suffering from a severe form of EB, five people assessed their condition as “mild,” and another five patients classified their severity as “moderate”.

### Limitations

This study presents several limitations. A first limitation is the small number of participants, a consequence arising from the rarity of the disease. Despite the team's efforts to include more participants, this was not possible due to the intervention's limited timeframe and the patients' difficulties attending. The small sample size (*n* = 14) represents a limitation of this study. Despite many efforts, it was not possible to include more participants, reflecting the rarity of the condition. In addition, the exploratory nature of this mixed-methods study limits the statistical generalisability of the findings, but allows a deeper understanding through a qualitative approach. Therefore, the small number of participants was counterbalanced by combining clinical assessment with quantitative and qualitative data in order to capture the diversity of individual situations. Most patients had RDEB, resulting in a limited range of EB types. This makes it impossible to perform any comparison between the EB types other than descriptive, as the DDEB group only presented one patient, and no JEB and KEB patients participated. However, given the considerable differences even within the same EB type, a broad range of severity, gender and age was opted for in the purposive sampling strategy to assess a diverse sample of teeth stages, dental cases and situations in Austria. While a bias might arise due to report by proxy for underage patients, the inclusion of diverse perspectives enabled a wider range of perspectives to be included. Sociodemographic information per participants were not included as the Austrian population with EB is very limited and data cannot be sufficiently de-identified even after anonymisation.

As a mixed-methods, cross-sectional study, this research aims to describe the findings observed in this cohort as an insight into the dental care situation for EB in Austria. To make these insights representative, larger studies are necessary to determine any causal relationship and to include a control group for comparison.

## Conclusions

People living with EB in Austria showed a diverse range of oral manifestations that impact their oral health-related quality of life (OHRQoL). Participants with RDEB presented lower levels of OHRQoL and EB-QoL, as well as worst oral health parameters and higher dental treatment needs. Oral function was clinically affected and compromise QoL especially in RDEB, but comprehensive dental treatment will not always improve oral function. Therefore, multidisciplinary oral health treatment including speech therapy and psychological support is needed for EB patients to improve not only their oral health, but also their OHRQoL. Future studies should analyse the effects of such multidisciplinary dental treatment on oral function and on OHRQoL.

## Data Availability

The original contributions presented in the study are included in the article/Supplementary Material, further inquiries can be directed to the corresponding authors.
