# Supplementary material for: Oral health status, treatment needs and oral health-related quality of life in patients with epidermolysis bullosa in Austria: a mixed-methods pilot study
Source: Front Oral Health. 2026 May 4;7:1826332. doi: 10.3389/froh.2026.1826332 (PMC13180850; doi:10.3389/froh.2026.1826332)
Supplement: Supplementary file 1 [file Table1.docx]

**SUPPLEMENTARY INFORMATION**

Supplementary figure 1: Flowchart of the study process.


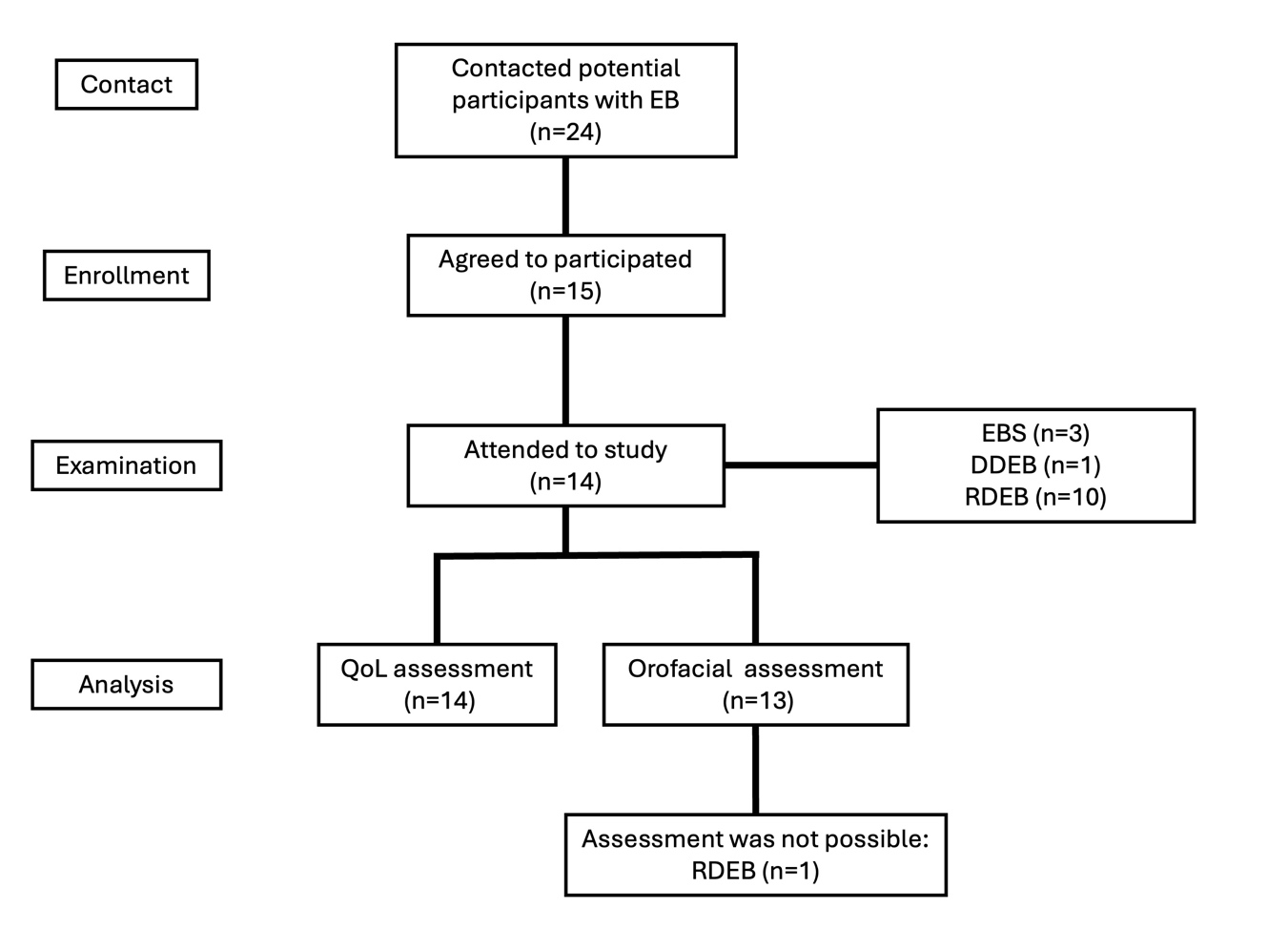


Appendix 1: Oral health assessment

The dental assessment was performed using the Epidermolysis Bullosa – Oral Health Assessment Form (EB-OHAF) from the Faculty of Dentistry of the University of Chile. The variables included in this study were:

- Extraoral lesions: Categorised by present/absent. Included the following conditions: Erythema, epithelial atrophy, milia, vesicles, blisters, bullae, ulcer, erosion, crust, scars, granulation tissue, angular cheilitis and lip atrophy.
- Intraoral lesions: Categorised by present/absent. Included the following conditions: Ulcer, erosion, vesicle, blister, bullae, blister in healing process, gingival hyperplasia non-plaque related, granulation tissue, atrophic tissue, scalloped tongue, fibrous scar bands and oral cancer.
- Mouth opening: Measured in mm in maximal mouth opening between the upper and lower incisors.
- Vestibule depth: Measured in mm with a periodontal probe from the bottom of the oral vestibule in resting position to the amelocemental junction (or alveolar ridge in case of teeth absence) in ten reference teeth (first molars, canines, and incisors level on the upper and lower arch).
- Tongue protrusion: Measured in mm from the incisal edge of the lower incisors to the tip of the tongue in a maximal protrusive movement.
- Tongue texture: Categorised as normal, partially depapillated or depapillated, assess the presence/absence of lingual papillae at the surface.
- Palatal texture: Categorised as normal, unusual or absent, assess the presence/absence of palatal rugae.
- Periodontal status: Periodontal assessment according to the Basic Periodontal Examination (BPE) Community Periodontal Index was assessed in each patient using a periodontal probe (PCP 12, Hu-Friedy) (44).
- DMFT: Assessment of Decay (D), Missing (M) and Filled (F) teeth for permanent and deciduous dentition.
- OHI: Oral Hygiene Index according to the Green and Vermillion(45)
- Malocclusion: Presence or absence or malocclusion, including crowding, , overjet or overbite over 4 mm, anterior or posterior crossbite and included tooth.
- Oral function: Presence or absence of mouth breathing, atypical deglutition, lingual interposition, lower lip interposition, food consistency modification, and history of oesophageal dilation.

Appendix 2: Oral treatment assessment

| Preventive  dentistry | Routine dental check-up, educational counselling, pits and fissures sealants, fluoride, and professional basic cleaning including plaque removal in periodontal pockets <3.5 mm (BPE:2). |
| --- | --- |
| Periodontics | Periodontal pockets >3.5 mm (BPE: 3 and 4), furcation involvement, periodontal defects or conditions that require periodontal surgery, patients with a medical condition not EB-related that affects the periodontal tissue. |
| Restorative | Caries or lesions (for example, hypoplasia) that require simple or complex filling without major oral rehabilitation (Decay from DFMT Index), including teeth that require endodontic treatment and later restorative treatment. |
| Orthodontics | Presence of malocclusions with aesthetic or functional impairment (severe crowding, anterior or posterior crossbite, severe increase in overjet or overbite, impacted or included teeth (except *8s), multidisciplinary management of spaces/occlusion). |
| Prosthodontics/ Implantology | Rehabilitation of missing teeth (Missing from DFMT Index), and teeth that require endodontic treatment with later major treatment such as crowns or bridges. |
| Surgery | Teeth extractions, orthognathic surgery, management of impacted or included teeth. |
| Speech Therapy | Functional anomalies including but not limited to the presence of mouth/ mixed breathing pattern, atypical deglutition, lingual interposition, lower lip interposition, food consistency modification, and history of oesophageal dilation. |
| Other | Any other professional that might be required to treat a specific condition. In this study, no patient needed a different specialty, therefore, was not included In the Table 2. |

BPE: Basic Periodontal Examination. DMFT: Decay-Missing-Filled Teeth index.
